# Complement protein levels and *MBL2* polymorphisms are associated with dengue and disease severity

**DOI:** 10.1038/s41598-020-71947-2

**Published:** 2020-09-10

**Authors:** Ngo Truong Giang, Hoang van Tong, Do Quyet, Nghiem Xuan Hoan, Trinh Huu Nghia, Nguyen Minh Nam, Hoang Vu Hung, Do Tuan Anh, Can Van Mao, Ho Anh Son, Christian G. Meyer, Thirumalaisamy P. Velavan, Nguyen Linh Toan

**Affiliations:** 1 Department of Pathophysiology, Vietnam Military Medical University, 160 Phung Hung, Ha Dong, Hanoi, Vietnam; 2 Department of Biology and Medical Genetics, Vietnam Military Medical University, Hanoi, Vietnam; 3 Institute of Biomedicine and Pharmacy, Vietnam Military Medical University, 222 Phung Hung, Ha Dong, Hanoi, Vietnam; 4 108 Military Central Hospital, Hanoi, Vietnam; 5 Vietnamese-German Center for Medical Research, VG-CARE, Hanoi, Vietnam; 6 103 Military Hospital, Vietnam Military Medical University, Hanoi, Vietnam; 7 Institute of Tropical Medicine, University of Tübingen, Tübingen, Germany; 8 Duy Tan University, Faculty of Medicine, Da Nang, Vietnam

**Keywords:** Genetics, Viral infection, Immunology, Immunogenetics, Immunopathogenesis, Infection

## Abstract

The complement system may be crucial during dengue virus infection and progression to severe dengue. This study investigates the role of *MBL2* genetic variants and levels of MBL in serum and complement proteins in Vietnamese dengue patients.* MBL2* genotypes (− 550*L/H, MBL2* codon 54*), MBL2* diplotypes (*XA/XO*, *YA/XO*) and *MBL2* haplotypes (*LXPB*, *HXPA*, *XO*) were associated with dengue in the study population. The levels of complement factors C2, C5, and C5a were higher in dengue and dengue with warning signs (DWS) patients compared to those in healthy controls, while factor D levels were decreased in dengue and DWS patients compared to the levels determined in healthy controls. C2 and C5a levels were associated with the levels of AST and ALT and with WBC counts. C9 levels were negatively correlated with ALT levels and WBC counts, and factor D levels were associated with AST and ALT levels and with platelet counts. In conclusions, *MBL2* polymorphisms are associated with dengue in the Vietnamese study population. The levels of the complement proteins C2, C4b, C5, C5a, C9, factor D and factor I are modulated in dengue patients during the clinical course of dengue.

## Introduction

Dengue is a mosquito-borne viral infection caused by dengue virus (DENV) with an estimated 390 million dengue infections worldwide every year and over 2.5 billion people at risk of the infection^1^. Dengue cases in South-East Asian countries are exponentially increasing with an annual average of 386,000 cases between 2001 and 2010^2^. Vietnam is a country with high dengue incidence and hyperendemic transmission of DENV. The outbreaks are generally larger and more common in southern provinces of Vietnam, with incidence peaking between June and October^3,4^.

Four distinct serotypes of the virus cause dengue (DENV-1, DENV-2, DENV-3 and DENV-4)^5^. DENVs circulate in Vietnam with a predominance of the DENV-1 and DENV-2 serotypes and DENV-2 appear to cause more severe conditions than the other serotypes^6^. The clinical manifestation of an acute DENV infection varies from mild febrile illness lasting approximately 4–7 days to life-threatening severe dengue haemorrhagic fever and dengue shock syndrome (DHF/DSS), characterized by plasma leakage, fluid accumulation, respiratory distress, severe bleeding, or organ impairment. The complex interplay of human innate and adaptive cellular immune responses with the virus is believed to influence the clinical course of DENV infection^7^. Recovery from infection provides lifelong immunity against the serotype which has caused the disease episode. However, cross-immunity to the other serotypes after recovery is only partial and short-term. Subsequent infections by other serotypes increase the risk of developing dengue haemorrhagic fever and such secondary infections are mediated by antibody-dependent immune enhancement (ADE)^7,8^.

Complement proteins, innate immune recognition molecules are activated either by classical, alternative or the lectin complement pathways during the early phase of infection^9^. The lectin complement pathway is triggered when the lectin proteins, namely the mannose-binding lectin (MBL) and/or the ficolins (FCN) bind to the pathogen recognition receptors on the surface of pathogens. Subsequently, a sequential enzyme cascade is triggered where MBL-associated protein 2 (MASP2) and other complement proteins (C5b-9) are activated leading to the formation of the membrane attack complex which eventually ends with opsonization, lysis, activation of inflammatory responses and/or clearance of immune complexes^9^. Human MBL encoded by *MBL2* gene is located on chromosome 10 and three single nucleotide polymorphisms (SNPs) in exon1 at codons 52 (MBL2*D), 54 (MBL2*B), and 57 (MBL2*C) respectively, interfere with the formation of higher MBL oligomers, causing alterations in functional activity of the protein and their circulating levels^10–12^. In addition, three promoter polymorphisms (positions, − 550, − 221 and + 4) were shown to regulate plasma MBL levels^12^. The MBL2*B, MBL2*C or MBL2*D mutant alleles are referred to as MBL2*O, whereas the wildtype is addressed as MBL2*A. MBL deficiency and *MBL2* polymorphisms have been shown to be associated with several infectious and autoimmune diseases^13,14^.

MBL was shown to neutralize DENV via complement-dependent and independent mechanisms, suggesting a significant role of MBL in the pathogenesis of dengue^15^. A previous study has indicated direct complement restriction of DENV infection via MBL recognition of the high-mannose glycans of DENV envelope proteins^16^. In addition, *MBL2* polymorphisms have been shown to be associated with dengue severity in Brazilian and Thai populations^17–19^. The above studies have investigated only the contribution of *MBL2* polymorphisms and MBL serum levels. The present study utilizing a Vietnamese study group has investigated not only MBL serum levels and *MBL2* variants but has also looked at associations of several downstream complement proteins of the complement pathway, namely C2, C4b, C5, C5a, C9, factor D and factor I with DENV infection.

## Materials and methods

### Study participants

A total of 279 unrelated Vietnamese patients (aged 34 ± 13; male/female, 130/149) with fever and symptoms of DENV infection admitted to the 103 Military Hospital of the Vietnam Military Medical University, Hanoi, collected between 2016 and 2018 were included. Patients were diagnosed based on the diagnostic criteria for dengue according to the World Health Organization (WHO)^20^. Patients were confirmed positive for NS1 antigen or/and positive for anti-DENV IgG and IgM as well as the clinical presentation of dengue. The patients were classified into two different groups according to the 2009 WHO dengue classification^20^. The first group included patients with dengue without warning signs (n = 172) and the second group comprised of dengue patients with warning signs (DWS, n = 107). DWS is characterized with fever, positive tourniquet test, petechiae or ecchymosis, maculopapular rash, myalgia/arthralgia, lymphadenopathy, leukopenia, thrombocytopenia and gastrointestinal bleeding with supportive serology of IgG and IgM titers and positive for NS1 antigen. Patients with dengue are usually characterized by a rapid and weak pulse (narrow pulse pressure < 20 mm Hg) with fever or a history of acute fever lasting 2–7 days (occasionally biphasic), haemorrhagic tendencies, evidenced by at least one of the following: a positive tourniquet test, petechiae, ecchymosis or purpura, bleeding from the mucosa, gastrointestinal tract, haematemesis or melaena^20^. Exclusion criteria were children below 12 and adults over 82 years, and patients who had any underlying chronic diseases and blood disorders. The demographic and clinical data of the study individuals are summarized in Table 1. Blood samples were collected on the day of admission and were used for measuring the levels of complement proteins. Serum samples were separated and stored at − 70 °C until further use. The control group included 200 healthy blood donors (aged 19.6 ± 1.2; male/female, 122/78) recruited in the same hospital and belonging to the Kinh ethnicity.

**Table 1:** Characteristics of study subjects.

Characteristics	Dengue (n = 172)	DWS (n = 107)	*P* value
**Age (years)**	31 (14–81)	31 (13–68)	NS
**Gender**			NS
Male n (%)	82 (47.7)	48 (44.9)	
Female n (%)	90 (52.3)	59 (55.1)	
**DENV NS1**			NS
Positive n (%)	153 (89.0)	93 (86.9)	
Negative n (%)	16 (9.3)	10 (9.3)	
Undetermined n (%)	3 (1.7)	4 (3.7)	
**DENV specific IgM**			NS
Positive n (%)	29 (16.9)	27 (25.2)	
Negative n (%)	138 (80.2)	75 (70.1)	
Undetermined n (%)	5 (2.9)	5 (4.7)	
**DENV specific IgG**			0.024
Positive n (%)	30 (17.4)	31 (29)	
Negative n (%)	137 (79.7)	71 (66.4)	
Undetermined n (%)	5 (2.9)	5 (4.7)	
**DENV serotype**			
DENV-1 n (%)	45 (26.2)	20 (18.7)	Reference
DENV-2 n (%)	8 (4.6)	13 (12.1)	0.019
DENV-1 and -2 n (%)	1 (0.6)	1 (0.9)	NS
Undetermined n (%)	118 (68.6)	69 (64.5)	
**DENV Viremia (1/Ct × 100)**	3 (2–6)^a^	3.1 (2–5)^b^	NS
**Day of illness at hospital admission**	5 (1–13)	5 (1–12)	NS
**Day of illness**	9 (4–20)	10 (6–16)	0.001
**Bleeding**			< 0.001
Yes n (%)	138 (80.2)	46 (43)	
No n (%)	34 (19.8)	61 (57)	
**Liver function test**			
AST (IU/ml)	61.3 (18–369.2)	108.3 (20.5–2,322)	< 0.001
ALT (IU/ml)	39 (8–301)	73.8 (8.6–992.3)	< 0.001
**Blood count test**			
RBC (× 10^3^/ml)	4.6 (2.8–6.81)	4.75 (2.88–7.17)	0.048
WBC (× 10^6^/ml)	3.39 (1–14.6)	3.38 (1.19–17.29)	NS
PLT (× 10^3^/ml)	86.5 (3.2–316)	60 (8–1,100)	< 0.001
HCT (%)	40 (27–53)	40.7 (24–52)	NS

Data presented as median (min–max).

*DENV* dengue virus; *DWS* dengue with warning signs; *NS1* non-structural antigen 1; *AST, ALT* aspartate and alanine amino transferase; *WBC* white blood cells; *RBC* red blood cells; *PLT* platelets; *HCT* haematocrit; *IU* international unit; *NS* not significant; *NA* not applicable.

^a^Done in 65 samples.

^b^Done in 35 samples.

### *MBL2* genotyping

Genomic DNA was extracted from blood samples using the PureLink Genomic DNA Mini Kit (Thermo Fisher Scientific, Waltham, MA, USA) following the manufacturer’s instruction. *MBL2* genotyping was performed as described previously^21^. Briefly, the *MBL2* promoter polymorphisms at − 550G>C, − 221G>C and 5′UTR + 4C>T and exon1 polymorphisms at codons 52C>T, 54G>A and 57G>A were amplified by PCR using primer pairs: PromF: 5′-GCC AGA AAG TAG AGA GGT ATT TAG CAC-3′, and Exon1R: 5′-CCA ACA CGT ACC TGG TTC CC-3′. The internal primer Exon1F: 5′-CAG GTG TCT AGG CAC AGA TGA ACC-3′ was used for Sanger sequencing of PCR products. The PCR assays were run in a 20 µl volume of reaction mixture containing 1× PCR buffer, 0.125 mM of dNTPs, 0.25 mM of each primer, 1U Taq DNA polymerase and 10 ng of genomic DNA. Thermal cycling conditions were: initial denaturation at 94 °C for 5 min, followed by 35 cycles of 30 s at 94 °C for denaturation, 30 s at 68 °C for annealing, 1 min 30 s at 72 °C for extension, followed by a final extension step of 2 min at 72 °C. PCR products were cleaned with Exo-SAP-IT and 1 µl of the purified product was used as templates for sequencing of the PCR products using the BigDye terminator v.1.1 (Applied Biosystems, Waltham, MA, USA) on an ABI 3130XL DNA sequencer. Polymorphisms were identified by assembling the sequences with reference sequences of the *MBL2* gene (NG_008196.1) using the Bioedit 7.2.6 software (https://www.mbio.ncsu.edu/BioEdit/bioedit.html) and were reconfirmed visually from their respective electropherograms.

### Quantification of plasma MBL and complement proteins

Levels of MBL and of the complement proteins C2, C4b, C5, C5a, C9, adipsin (factor D) and factor I were measured in serum samples from patients and healthy individuals using the MILLIPLEX MAP human Complement Magnetic Bead Panel 1 (Millipore Corporation, Billerica, MA, USA; Cat. Number: HCMP1MAG-19K), according to the manufacturer’s instructions. Briefly, after washing the plate with washing buffer, 25 μl of each diluted standard, control and diluted plasma samples (1:100) were added to the wells and 25 μl of assay buffer were added to the sample wells. 25 μl of the mixed beads were added to each well; the plate was covered with a sealer and wrapped with foil and then incubated with agitation on a plate shaker overnight at 2–8 °C. The well contents were removed and the plates were washed three times. Subsequently, 50 μl of detection antibodies were added to each well. Plates were sealed and incubated under agitation for 1 h at ambient temperature. 50 μl of streptavidin–phycoerythrin were added to each well and the sealed plates were incubated again on a shaker for 30 min at room temperature. Next, the well contents were removed and plates were washed three times. 150 μl of sheath fluid were added to all wells and the beads were resuspended on a plate shaker for 5 min. Plates were subjected to a Luminex 200 system and median fluorescent intensity (MFI) data were analyzed using a 5-parameter logistic method for calculating protein concentrations in the samples.

### Statistical analysis

Quantitative variables were compared using Kruskal–Wallis or Mann–Whitney U tests and were given as medians with range. Categorical variables were compared using Chi-square or Fisher’s exact tests and given as percentages. Genotype and allele frequencies were analyzed by simple gene counting. *MBL2* haplotypes were analysed using the expectation-maximum (EM) algorithm implemented in the Arlequin v. 3.5.1.2 software (https://cmpg.unibe.ch/software/arlequin3512/) and deviations from Hardy–Weinberg (HW) equilibrium were tested. Linkage disequilibrium (LD) analysis was performed using the Haploview v. 3.2 program (https://www.broadinstitute.org/haploview/haploview). Binary logistic regression model adjusted for confounding factors (age and gender) was used to analyze the association of *MBL2* polymorphisms (genotypes, alleles, haplotypes and diplotypes) with dengue fever and disease severity. Odds ratios (ORs) and their 95% confidence intervals (CIs) were calculated. Kruskal–Wallis test or Mann–Whitney U test was used to analyze the association of serum MBL levels with *MBL2* gene variants. All statistical analyses were performed with Stata v.14 (StataCorp, College Station, Texas, USA) and SPSS software version 22.0 (SPSS Statistics, IBM, Armonk, NY, USA) and the level of significance was set to a *P* value of < 0.05.

### Ethics statement

Patients and healthy individuals were informed in detail about the study and written consent was given by all participants or by a parent of patients if the patients were under 18 years old. All clinical procedures and experiments were performed in accordance with international and national guidelines. The study was approved by the Institutional Review Board of the Vietnam Military Medical University, Hanoi, Vietnam (103MCH/RES/DENV-GER_V-D1-2016).

## Results

### Baseline characteristics of study groups

**Figure 1:**
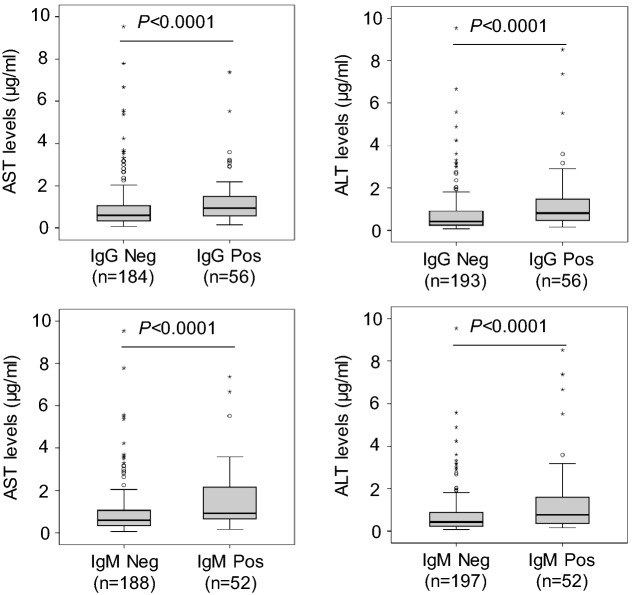
Liver enzyme levels in patients positive and negative with DENV-specific antibodies. *Pos* positive, *Neg* negative. *P* values were calculated by Mann–Whitney *U* test.

Demographic and clinical characteristics of dengue patients are summarized in Table 1. There was no difference of mean age, gender distribution, positivity rate of DENV NS1 and DENV-specific IgM positivity between the dengue and DWS groups. The DENV-specific IgG positivity rate was significantly higher in the DWS compared to the dengue group (*P* = 0.024). The DENV-1 serotype was predominant in the study cohort and DENV-2 was observed more frequently in DWS patients compared to dengue patients (*P* = 0.019) indicating the association of DENV-2 with more severity. No significant difference in DENV viremia was observed. Days of illness at hospital admission were not different between dengue and DWS patients (*P* > 0.05). Nevertheless, days of illness (from onset of symptoms to hospital discharge) in DWS patients were longer compared to dengue patients (*P* = 0.001). The levels of AST and ALT were significantly increased in patients with DWS compared to dengue patients (*P* < 0.001), indicating that DWS is associated with increased liver injuries. Although the HCT and WBC and WBC counts did not differ between the dengue and DWS groups, the platelet counts were significantly lower in patients with DWS compared to those with dengue (*P* < 0.001) (Table 1). We compared AST and ALT levels between patient subgroups positive and negative for DENV-specific IgG and IgM antibodies. AST and ALT levels were significantly increased in patients positive for DENV-specific IgG and IgM compared to those negative for DENV-specific IgG and IgM (*P* < 0.0001) (Fig. 1). These results indicate that DENV infection is associated with liver injury.

### Association of *MBL2* polymorphisms with dengue

The genotype frequencies of *MBL2* SNPs in the control group were in Hardy–Weinberg equilibrium (*P* > 0.05). The distribution of *MBL2* genotype and allele frequencies is shown in Table 2. SNPs in the promoter region were in strong linkage disequilibrium both among patients and controls. The frequency of the promoter allele − *550H* was significantly higher in controls compared to all dengue patients (dengue and DWS), suggesting a relative protective effect (OR = 0.6, 95%CI = 0.37–0.95, *P* = 0.03). The frequency of the minor allele MBL2*A at codon 54 was significantly higher in all dengue patients (dengue and DWS) compared to controls (dengue plus DWS: OR = 2.1, 95% CI 1.2–3.8, *P* = 0.012; dengue: OR = 1.99, 95% CI 1.0–4.1, *P* = 0.05; DWS: OR = 2.2, 95% CI 1.05–4.8, *P* = 0.037), indicating that MBL2*A at codon 54 may confer an increased risk of dengue infection. Similarly, the frequencies of allele MBL2*O at exon1 (codons 52 + 54 + 57) were also higher among all patients (dengue plus DWS) compared to healthy controls (dengue plus DWS: OR = 1.97, 95% CI 1.1–3.5, *P* = 0.021; DWS: OR = 2.1, 95% CI 1.0–4.5, *P* = 0.05), indicating an increased risk of dengue (Table 2). No significant differences were observed for other *MBL2* variants (− 221, + 4, codons 52 and 57) in comparisons of dengue patients with controls. We observed the variant at codon 52 with a low allele frequency (1.4%) in the control group but not in the patient group. The variant at codon 57 was not observed in our study population. Furthermore, there was no significant difference in genotype and allele frequencies of *MBL2* variants in the comparison of dengue with DWS patients.

**Table 2:** Genotype and allele distribution of *MBL2* polymorphisms in dengue patients and healthy controls.

*MBL2* polymorphisms	All patients n (%)	Dengue n (%)	DWS n (%)	Healthy controls n (%)	All patients vs. healthy controls		Dengue vs. healthy controls		DWS vs. healthy controls	
					OR (95% CI)	*P* value	OR (95% CI)	*P* value	OR (95% CI)	*P* value
**rs11003125 (− 550C/G)**										
*CC (LL)*	77 (33.0)	53 (37.3)	24 (26.4)	40 (23.5)		Reference		Reference		Reference
*GC (HL)*	95 (40.8)	52 (36.6)	43 (47.3)	76 (44.7)		NS		NS		NS
*GG (HH)*	61 (26.2)	37 (26.1)	24 (26.4)	54 (31.8)		NS		NS		NS
*C (L)*	249 (53.4)	158 (55.6)	91 (50)	156 (45.9)		Reference		Reference		Reference
*G (H)*	217 (46.6)	126 (45.4)	91 (50)	184 (54.1)	**0.6 (0.37–0.95)**	**0.03**		NS		NS
**rs7096206 (− 221 G/C,A)**										
*CC (XX)*	152 (56.5)	97 (59.1)	55 (52.4)	99 (55.6)		Reference		Reference		Reference
*CG(A) (XY)*	94 (34.9)	53 (32.3)	41 (39.0)	66 (37.1)		NS		NS		NS
*GG (YY)*	23 (8.6)	14 (8.5)	9 (8.6)	13 (7.3)		NS		NS		NS
*C (X)*	398 (74)	247 (75.3)	151 (71.9)	264 (74.9)		Reference		Reference		Reference
*G (Y)*	140 (26)	81 (24.7)	59 (28.1)	92 (25.1)		NS		NS		NS
**rs7095891 (+ 4G/A)**										
*GG (PP)*	233 (84.1)	143 (83.6)	90 (84.9)	169 (86.2)		Reference		Reference		Reference
*GA (PQ)*	42 (15.1)	28 (16.4)	14 (13.1)	25 (12.8)		NS		NS		NS
*AA (QQ)*	2 (0.7)	0 (0)	2 (1.9)	2 (1.0)		NS		NS		NS
*G (P)*	508 (91.7)	314 (91.8)	194 (91.5)	363 (92.6)		Reference		Reference		Reference
*A (Q)*	46 (8.3)	28 (8.2)	18 (8.5)	29 (7.4)		NS		NS		NS
MBL2*Exon1 (codon 54) G>A										
*GG*	161 (59.7)	96 (58.2)	64 (62.1)	140 (78.2)		Reference		Reference		Reference
*GA*	103 (38.8)	67 (40.6)	37 (35.9)	38 (21.2)	**2.6 (1.4–5.1)**	**0.004**	**2.4 (1.1–5.5)**	**0.034**	**2.9 (1.2–6.9)**	**0.018**
*AA*	4 (1.5)	2 (1.2)	2 (1.9)	1 (0.6)		NS		NS		NS
*G*	425 (79.3)	260 (78.8)	165 (80.1)	318 (88.8)		Reference		Reference		Reference
*A*	111 (20.7)	70 (21.2)	41 (19.9)	40 (11.2)	**2.1 (1.2–3.8)**	**0.012**	**1.99 (1–4.1)**	**0.05**	**2.2 (1.05–4.8)**	**0.037**
MBL2*Exon1 codons 52 + 54 + 57										
*AA*	161 (59.7)	96 (58.2)	64 (62.1)	137 (76.5)		Reference		Reference		Reference
*AO*	103 (38.8)	67 (40.6)	37 (35.9)	41 (22.9)	**2.4 (1.3–4.7)**	**0.008**	**2.2 (1.0 − 5.1)**	**0.05**	**2.7 (1.1–6.4)**	**0.027**
*OO*	4 (1.5)	2 (1.2)	2 (1.9)	1 (0.6)		NS		NS		NS
*A*	425 (79.3)	260 (78.8)	165 (80.1)	315 (88)		Reference		Reference		Reference
*O*	111 (20.7)	70 (21.2)	41 (19.9)	43 (12)	**1.97 (1.1–3.5)**	**0.021**		NS	**2.1 (1–4.5)**	**0.05**

Bold values indicate statistical significance.

*DWS* dengue with warning signs, *NS* not significant.

We reconstructed *MBL2* haplotypes based on the − *221Y/X* promoter variant and three structural exon1 SNPs (MBL2*A/O), which mainly regulate MBL serum levels. The frequency of the diplotype *XA/XO* was significantly higher in patients compared to healthy controls (dengue plus DWS: OR = 3.3, 95% CI 1.4–8.1, *P* = 0.007; dengue: OR = 3.1, 95% CI 1.0–9.3, *P* = 0.044; DWS: OR = 3.7, 95% CI 1.1–12.1, *P* = 0.029), suggesting an increased risk of dengue fever. In addition, the frequency of haplotype *XO* was significantly higher in patients compared to healthy controls (dengue plus DWS: OR = 2.2, 95% CI 1.2–4.2, *P* = 0.015; DWS: OR = 2.7, 95% CI 1.2–5.9, *P* = 0.015). There was no significant difference in the comparison of dengue with DWS patients (Table 3).

**Table 3:** *MBL2* diplotypes in dengue patients and healthy controls.

*MBL2 *(− 221) + Exon1	All patients	Dengue	DWS	Healthy controls	All patients vs. healthy controls		Dengue vs. healthy controls		DWS vs. healthy controls	
	n (%)	n (%)	n (%)	n (%)	OR (95% CI)	*P* value	OR (95% CI)	*P* value	OR (95% CI)	*P* value
**Diplotypes**	n = 260	n = 158	n = 102	n = 161						
*XA/XA*	82 (31.5)	52 (32.9)	30 (29.4)	68 (42.2)		Reference		Reference		Reference
*XA/XO*	59 (22.7)	38 (24.1)	21 (20.6)	21 (13.0)	**3.3 (1.4–8.1)**	**0.007**	**3.1 (1.0–9.3)**	**0.044**	**3.7 (1.1–12.1)**	**0.029**
*XA/YA*	58 (22.4)	33 (20.9)	25 (24.5)	46 (28.6)		NS		NS		NS
*YA/XO*	35 (13.5)	20 (12.7)	15 (14.7)	13 (8.1)		NS		NS	**3.8 (1–14.9)**	**0.05**
*YA/YA*	19 (7.3)	11 (7.0)	8 (7.8)	10 (6.2)		NS		NS		NS
*YA/YO*	3 (1.2)	2 (1.3)	1 (1.0)	2 (1.2)		NS		NS		NA
*XO/XO*	4 (1.5)	2 (1.3)	2 (2.0)	1 (0.6)		NS		NS		NS
**Haplotype**	n = 520	n = 316	n = 204	n = 322						
*XA*	281 (54.0)	175 (55.4)	106 (52.0)	203 (63.0)		Reference		Reference		Reference
*YA*	134 (25.8)	77 (24.4)	57 (27.9)	81 (25.2)		NS		NS		NS
*XO*	102 (19.6)	62 (19.6)	40 (19.6)	36 (11.2)	**2.2 (1.2–4.2)**	**0.015**		NS	**2.7 (1.2–5.9)**	**0.015**
*YO*	3 (0.6)	2 (0.6)	1 (0.5)	2 (0.6)		NS		NS		NS

Bold values indicate statistical significance.

*DWS* dengue with warning signs, *NS* not significant, *NA* not applicable.

We reconstructed *MBL2* haplotypes based on three promoter SNPs and three exon 1 SNPs. The frequency of the haplotype *LXPB* was significantly higher in patients compared to controls (dengue plus DWS: OR = 4.1, 95% CI 1.2–14.3, *P* = 0.027; DWS: OR = 5.6, 95% CI 1.2–26, *P* = 0.028), suggesting an increased risk of dengue fever. The *HXPA* haplotype occurred less frequently among patients compared with controls (dengue plus DWS: OR = 0.3, 95% CI 0.1–0.9, *P* = 0.025; DWS: OR = 0.2, 95% CI 0.03–0.98, *P* = 0.047), suggesting a protective role in DENV infection. However, no significant difference in haplotype frequencies was observed between dengue and DWS patients (Table 4).

**Table 4:** *MBL2* haplotypes in dengue patients and healthy controls.

*MBL2* haplotypes	All patients	Dengue	DWS	Healthy controls	All patients vs. healthy controls		Dengue vs. healthy controls		DWS vs. healthy controls	
	n = 452 (%)	n = 274 (%)	n = 176 (%)	n = 276 (%)	OR (95% CI)	*P* value	OR (95% CI)	*P* value	OR (95% CI)	*P* value
*LXPA*	201 (44.7)	127 (46.4)	74 (42.0)	120 (43.5)		Reference		Reference		Reference
*HYPA*	116 (25.8)	67 (24.5)	49 (27.8)	70 (25.4)		NS		NS		NS
*HXPB*	62 (13.8)	37 (13.5)	25 (14.2)	23 (8.3)		NS		NS		NS
*LXPB*	29 (6.4)	19 (6.9)	10 (5.7)	6 (2.2)	**4.1 (1.2–14.3)**	**0.027**		NS	**5.6 (1.2–26)**	**0.028**
*HXPA*	24 (5.3)	16 (5.8)	8 (4.5)	39 (14.1)	**0.3 (0.1–0.9)**	**0.025**		NS	**0.2 (0.03–0.98)**	**0.047**
*HXQA*	5 (1.1)	1 (0.4)	4 (2.3)	6 (2.2)		NS		NS		NS
*LXQA*	6 (1.3)	3 (1.1)	3 (1.7)	4 (1.4)		NS		NS		NS
*LYPA*	4 (0.9)	2 (0.7)	2 (1.1)	3 (1.1)		NS		NS		NS
*HYPB*	3 (0.7)	2 (0.7)	1 (0.6)	0 (0.0)		NA		NA		NA
*LYPB*	0 (0.0)	0 (0.0)	0 (0.0)	2 (0.7)		NA		NA		NA
*HXPD*	0 (0.0)	0 (0.0)	0 (0.0)	2 (0.7)		NA		NA		NA
*LXPD*	0 (0.0)	0 (0.0)	0 (0.0)	1 (0.4)		NA		NA		NA

Bold values indicate statistical significance.

*DWS* dengue with warning signs, *NS* not significant, *NA* not applicable.

### Serum levels of MBL and complement proteins in patients with Dengue

**Figure 2:**
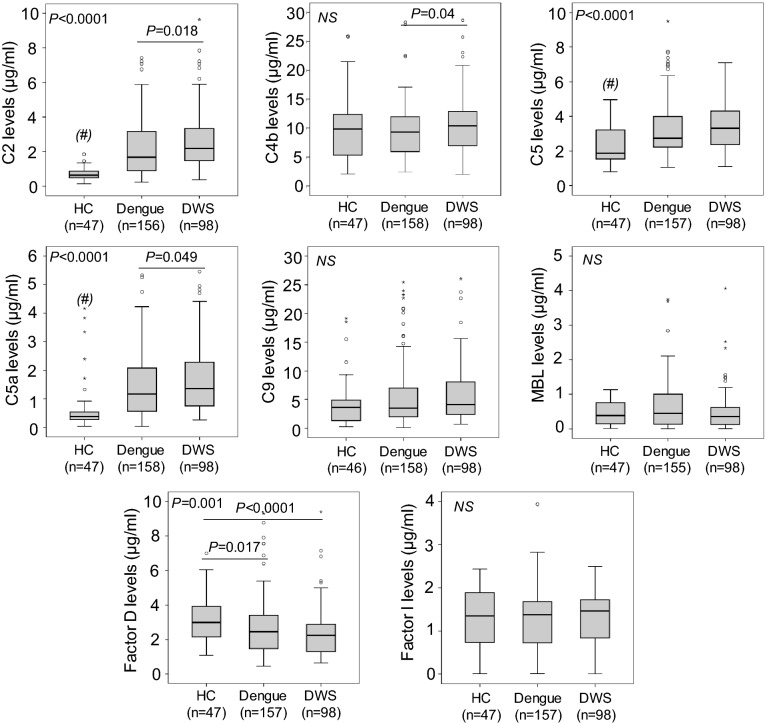
Complement protein levels in dengue patients and healthy individuals. *HC* healthy control, *DWS* dengue patients with warning signs. *P* values were calculated by Kruskal–Wallis test or Mann–Whitney *U* test, where appropriate.

We measured the levels of MBL and the complement proteins C2, C4b, C5, C5a, C9, adipsin (factor D) and factor I in serum samples of dengue and DWS patients and healthy controls. The levels of C2, C5, and C5a were significantly higher in dengue and DWS patients compared to those in healthy controls (*P* < 0.0001) and also higher in DWS patients compared to those in dengue patients (*P* = 0.018, < 0.0001 and 0.049, respectively). The levels of factor D were decreased in dengue and DWS patients compared to those in controls (*P* = 0.017 and < 0.0001, respectively). No difference of the levels of C4b, C9, MBL and factor I was observed among dengue and DWS patients and healthy controls (*P* > 0.05) (Fig. 2). These results indicate that the levels of C2, C5, C5a and factor D are modulated during DENV infection and progression of dengue.

**Figure 3:**
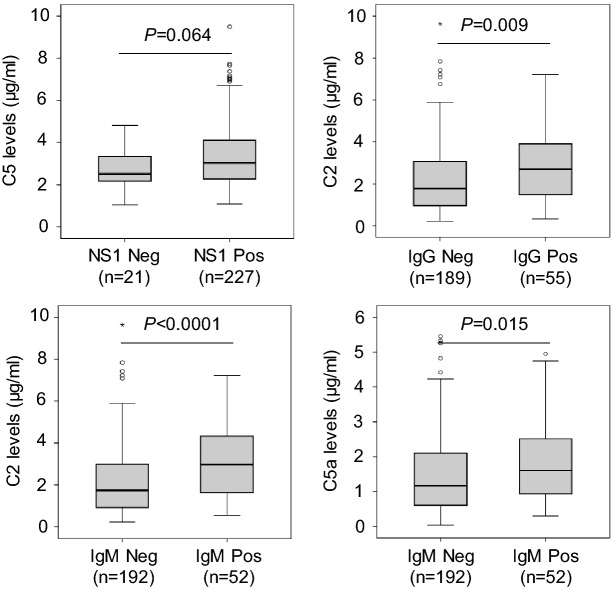
Complement protein levels in patients positive and negative with DENV-specific antibodies. *NS1* non-structural antigen 1, *Pos* positive, *Neg* negative. *P* values were calculated by Mann–Whitney *U* test.

We then segregated the patients into subgroups based on positivity for NS1 and DENV-specific IgG and IgM antibodies and compared the levels of C2, C4b, C5, C5a, C9, factor D and factor I between these subgroups. C5 levels were marginally increased in patients positive compared to those negative for NS1 (*P* = 0.064). C2 levels were increased in IgG positive patients compared to those negative for IgG antibodies (*P* = 0.0009). The levels of C2 and C5a were significantly increased in IgM positive compared to patients negative for IgM (*P* < 0.0001 and *P* = 0.015, respectively) (Fig. 3). These results indicate that the levels of C2, C5 and C5a might be associated with DENV infection. In addition, we classified the patients into subgroups based on the infection with DENV-1 or DENV-2 and compared the levels of C2, C4b, C5, C5a, C9, factor D and factor I between these classified groups. However, no statistical significance was observed.

### Association of *MBL2* polymorphisms with MBL serum levels

**Figure 4:**
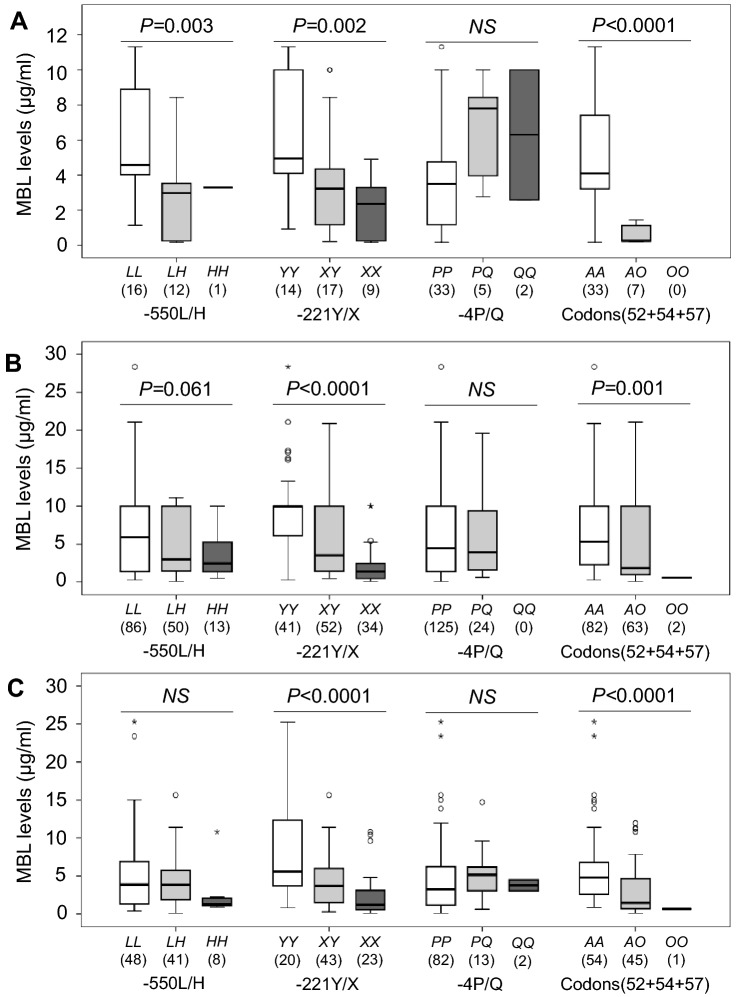
Distribution of MBL levels in individuals with different *MBL2* genotypes. (**A**), healthy individuals; (**B**), dengue patients; (**C**), dengue patients with warning signs (DWS). *P* values were calculated by Kruskal–Wallis test or Mann–Whitney *U* test, where appropriate.

The association of *MBL2* polymorphisms with MBL levels has been established in different world population. Therefore, we analyzed the distribution of MBL serum levels across different *MBL2* genotypes and haplotypes. Alleles MBL2*L and MBL2*Y at the promoter region and allele MBL2*A at codons 52 + 54 + 57 significantly contributed to higher MBL levels, while alleles MBL2*H MBL2*X and MBL2*O contributed to lower MBL levels in healthy individuals (*P* < 0.05). *MBL2 *− 4P/Q polymorphism was not significantly associated with MBL levels in healthy individuals (Fig. 4A). In dengue and DWS patients, allele MBL2*Y at the promoter region and allele MBL2*A at codons 52 + 54 + 57 significantly contributed to higher MBL levels, whereas alleles MBL2*X and MBL2*O contributed to lower MBL levels (*P* < 0.05). However, significant associations of *MBL2 *− 550L/H and *MBL2 *− 4P/Q polymorphisms with MBL levels in dengue and DWS patients were not observed (*P* < 0.05) (Fig. 4B,C).

### Correlation of serum levels of complement proteins with clinical parameters

The correlation between a pair of complement protein levels as well as between complement protein levels and clinical parameters in dengue patients was analyzed. In healthy individuals, C2 levels were significantly associated with C4b, C5, C5a and C9 levels (Spearman’s rho = 0.49, 0.7, 0.39, and 0.3; *P* = 0.0005, < 0.0001, 0.006, and 0.046, respectively). C4b levels were correlated with the levels of C5, C5a, C9 and factor I (Spearman’s rho = 0.64, 0.37, 0.42 and 0.29; *P* < 0.0001, 0.011, 0.004, and 0.049, respectively). C5 levels were associated with C5a and factor I levels (Spearman’s rho = 0.52 and 0.35; *P* = 0.0002 and 0.016, respectively) (Table 5).

**Table 5:** Correlation of complement proteins in healthy individuals.

	C2 (µg/ml)	C4b (µg/ml)	C5 (µg/ml)	C5a (µg/ml)	C9 (µg/ml)	Factor D (µg/ml)	MBL (µg/ml)	Factor I (µg/ml)
**C2 (µg/ml)**								
*r*_*s*_		**0.49**	**0.7**	**0.39**	**0.3**	0.14	− 0.17	0.13
*P*		**0.0005**	**< 0.0001**	**0.006**	**0.046**	0.35	0.26	0.37
**C4b (µg/ml)**								
*r*_*s*_	**0.49**		**0.64**	**0.37**	**0.42**	0.013	− 0.002	**0.29**
*P*	**0.0005**		**< 0.0001**	**0.011**	**0.004**	0.93	0.99	**0.049**
**C5 (µg/ml)**								
*r*_*s*_	**0.7**	**0.64**		**0.52**	0.27	0.09	− 0.12	**0.35**
*P*	**< 0.0001**	**< 0.0001**		**0.0002**	0.07	0.56	0.41	**0.016**
**C5a (µg/ml)**								
*r*_*s*_	**0.39**	**0.37**	**0.52**		0.15	0.079	0.09	0.2
*P*	**0.006**	**0.011**	**0.0002**		0.31	0.6	0.53	0.18
**C9 (µg/ml)**								
*r*_*s*_	**0.3**	**0.42**	0.27	0.15		− 0.18	0.004	0.15
*P*	**0.046**	**0.004**	0.07	0.31		0.24	0.98	0.31
**Factor D (µg/ml)**								
*r*_*s*_	0.14	0.013	0.09	0.079	− 0.18		0.15	0.011
*P*	0.35	0.93	0.56	0.6	0.24		0.32	0.94
**MBL (µg/ml)**								
*r*_*s*_	− 0.17	− 0.002	− 0.12	0.09	0.004	0.15		− 0.15
*P*	0.26	0.99	0.41	0.53	0.98	0.32		0.32
**Factor I (µg/ml)**								
*r*_*s*_	0.13	**0.29**	**0.35**	0.2	0.15	0.011	− 0.15	
*P*	0.37	**0.049**	**0.016**	0.18	0.31	0.94	0.32	

Spearman’s rho (r_s_) calculated by Spearman's rank correlation coefficient. Bold r_s_ and *P* values indicate a significant and strong correlation between variables.

In dengue patients, C2 levels correlated with the levels of C4b, C5, C5a and factor D (Spearman’s rho = 0.5, 0.39, 0.61, − 0.25; *P* < 0.0001, < 0.0001, < 0.0001 and = 0.0002, respectively). C4b levels correlated with C5 and C5a levels (Spearman’s rho = 0.52, 0.38; *P* < 0.0001, respectively). Factor D levels were negatively associated with C5a levels (Spearman’s rho = − 0.34; *P* < 0.0001), while they positively correlated with C9 levels (Spearman’s rho = 0.21; *P* = 0.01). Regarding the correlation between complement protein levels and clinical parameters, C4b levels were significantly associated with platelet counts (Spearman’s rho = 0.25; *P* = 0.002), and factor D levels were positively correlated with RBC counts and HCT values (Spearman’s rho = 0.31, 0.32; *P* < 0.0001, respectively), while negatively correlated with WBC and platelet counts (Spearman’s rho = − 0.24, − 0.31; *P* = 0.002 and < 0.0001, respectively) (Table 6, upper-right area).

**Table 6:** Correlation of complement proteins with clinical parameters in dengue patients.

	C2 (µg/ml)	C4b (µg/ml)	C5 (µg/ml)	C5a (µg/ml)	C9 (µg/ml)	Factor D (µg/ml)	MBL (µg/ml)	Factor I (µg/ml)	AST (IU/ml)	ALT (IU/ml)	RBC (× 10^3^/ml)	WBC (× 10^6^/ml)	PLT (× 10^3^/ml)	HCT (%)
**C2 (µg/ml)**														
*r*_*s*_		**0.5**	**0.39**	**0.61**	0.052	**− 0.25**	0.004	0.02	0.14	0.13	− 0.09	0.16	0.07	− 0.12
*P*		**< 0.0001**	**< 0.0001**	**< 0.0001**	0.51	**0.002**	0.96	0.79	0.09	0.11	0.27	0.04	0.38	0.14
**C4b (µg/ml)**														
*r*_*s*_	**0.35**		**0.52**	**0.38**	0.08	− 0.15	− 0.09	0.19	− 0.1	− 0.04	− 0.15	0.06	**0.25**	− 0.12
*P*	**< 0.0001**		**< 0.0001**	**< 0.0001**	0.29	0.06	0.23	0.014	0.22	0.66	0.053	0.45	**0.002**	0.15
**C5 (µg/ml)**														
*r*_*s*_	0.17	**0.37**		**0.37**	**0.32**	0.04	− 0.11	0.12	0.03	0.003	0.03	0.09	0.03	0.03
*P*	0.08	**< 0.0001**		**< 0.0001**	**< 0.0001**	0.58	0.16	0.45	0.69	0.97	0.72	0.23	0.76	0.76
**C5a (µg/ml)**														
*r*_*s*_	**0.62**	**0.32**	**0.34**		− 0.17	**− 0.34**	0.07	− 0.06	0.16	0.1	0.02	0.06	− 0.03	0.01
*P*	**< 0.0001**	**0.001**	**0.001**		0.03	**< 0.0001**	0.39	0.46	0.64	0.23	0.81	0.43	0.67	0.9
**C9 (µg/ml)**														
*r*_*s*_	**− 0.24**	− 0.14	0.18	**− 0.31**		**0.21**	− 0.05	0.12	− 0.044	− 0.001	0.015	− 0.02	0.09	0.005
*P*	**0.02**	0.18	0.08	**0.002**		**0.01**	0.51	0.12	0.6	0.99	0.86	0.79	0.26	0.95
**Factor D (µg/ml)**														
*r*_*s*_	**− 0.5**	**− 0.23**	0.09	**− 0.53**	**0.49**		− 0.17	0.145	0.09	0.06	**0.31**	**− 0.24**	**− 0.31**	**0.32**
*P*	**< 0.0001**	**0.03**	0.39	**< 0.0001**	**< 0.0001**		0.034	0.69	0.34	0.61	**< 0.0001**	**0.002**	**< 0.0001**	**< 0.0001**
**MBL (µg/ml)**														
*r*_*s*_	0.18	0.06	0.11	0.16	− 0.17	− 0.12		− 0.04	− 0.01	0.01	− 0.08	− 0.05	0.11	− 0.02
*P*	0.07	0.54	0.29	0.11	0.09	0.23		0.59	0.9	0.94	0.31	0.58	0.17	0.71
**Factor I (µg/ml)**														
*r*_*s*_	0.12	**0.29**	**0.24**	− 0.05	0.09	**0.25**	0.06		− 0.13	− 0.15	− 0.08	− 0.005	0.15	− 0.1
*P*	0.23	**0.004**	**0.02**	0.62	0.38	**0.01**	0.53		0.14	0.06	0.34	0.95	0.065	0.24
**AST (IU/ml)**														
*r*_*s*_	**0.27**	0.07	− 0.02	**0.22**	− 0.18	**− 0.25**	− 0.12	0.02		**0.87**	**0.28**	− 0.03	**− 0.46**	**0.23**
*P*	**0.012**	0.54	0.88	**0.04**	0.09	**0.02**	0.29	0.8		**< 0.0001**	**< 0.0001**	0.75	**< 0.0001**	**0.005**
**ALT (IU/ml)**														
*r*_*s*_	**0.32**	0.14	− 0.001	**0.3**	**− 0.27**	**− 0.25**	− 0.03	0.04	**0.91**		**0.27**	0.09	**− 0.34**	**0.21**
*P*	**0.002**	0.2	0.99	**0.005**	**0.01**	**0.015**	0.8	0.7	**< 0.0001**		**0.001**	0.27	**< 0.0001**	**0.008**
**RBC (× 10**^**3**^**/ml)**														
*r*_*s*_	0.11	**− 0.22**	0.03	0.03	0.04	0.18	0.02	0.05	0.001	0.07		0.006	**− 0.3**	**0.87**
*P*	0.28	**0.03**	0.79	0.8	0.7	0.08	0.85	0.66	0.99	0.5		0.94	**< 0.0001**	**< 0.0001**
**WBC (× 10**^**6**^**/ml)**														
*r*_*s*_	**0.24**	0.04	0.02	**0.25**	**− 0.23**	− 0.16	0.18	**0.25**	**0.26**	**0.36**	**0.25**		**0.26**	0.04
*P*	**0.02**	0.7	0.86	**0.012**	**0.02**	0.11	0.07	**0.014**	**0.013**	**< 0.0001**	**0.009**		**< 0.0001**	0.61
**PLT (× 10**^**3**^**/ml)**														
*r*_*s*_	− 0.08	0.14	0.11	0.05	− 0.05	**− 0.22**	− 0.07	− 0.05	− 0.1	− 0.007	**− 0.26**	− 0.09		**− 0.28**
*P*	0.43	0.14	0.29	0.6	0.6	**0.026**	0.5	0.63	0.32	0.94	**0.006**	0.36		**< 0.0001**
**HCT (%)**														
*r*_*s*_	0.004	− 0.24	0.03	− 0.07	− 0.04	0.18	− 0.02	0.05	0.04	0.08	**0.86**	**0.26**	**− 0.32**	
*P*	0.97	0.17	0.78	0.68	0.68	0.067	0.87	0.6	0.7	0.4	**< 0.0001**	**0.006**	**0.001**	

Spearman’s rho (r_s_) calculated by Spearman's rank correlation coefficient. r_s_ and *P* values in the upper-right area are the correlation between variables in dengue patients. r_s_ and *P* values in the lower-left area are the correlation between variables in dengue patients with warning signs (DWS).

Bold r_s_ and *P* values indicate a significant and strong correlation between variables.

*AST and ALT* aspartate and alanine amino transferase, *WBC* white blood cells, *RBC* red blood cells, *PLT* platelets, *HCT* haematocrit, *IU* international unit.

In DWS patients, the correlation patterns differed from those observed in dengue patients. In particular, C2 levels were significantly associated with C4b, C5a, C9 and factor D levels. C4b levels were significantly correlated with the levels of C5, C5a, factor D, and factor I. C5 levels significantly correlated with C5a and factor I levels and factor D levels were associated with the C9 and factor I levels. With regard to correlations of complement proteins with laboratory parameters, C2 and C5a levels significantly correlated with AST and ALT levels, and with WBC counts. C9 levels were negatively correlated with ALT levels and WBC counts. Factor D levels were negatively correlated with AST and ALT levels and platelet counts. A negative correlation between C4b levels and RBC counts and a positive correlation between factor I levels and WBC counts was also observed (Table 6, lower-left area).

## Discussion

Dengue is a serious health problem in tropical and subtropical areas including Vietnam. Studies have shown that the pathogenesis and severity of the disease are closely associated with distinct host immune responses against DENV, including components of the complement system^22,23^. Here we show that *MBL2* polymorphisms and the levels of the complement proteins C2, C4b, C5, C5a, C9, factor D and factor I are associated with DENV infection and severity of the disease. The results indicate that complement proteins are modulated during DENV infection and play an important role in the innate immune response against DENV infection and in the progression to severe dengue.

The association of *MBL2* polymorphisms with infectious diseases has previously been documented^13,14^. In particular, the MBL2*OO genotype, the MBL2*O allele and *MBL2* haplotypes, which correlate with low MBL levels have been shown to be associated with DHF in a Brazilian study group^18^. A recent study has also shown an association of *MBL2* haplotypes with an increased risk of dengue severity in Brazilians^19^. However, no association between *MBL2* polymorphisms and DENV infection was observed in a Thai study group^24^. A genome-wide association study has identified SNP rs3132468 in the MHC class I polypeptide-related sequence B (*MICB*) and SNP rs3765524 in the phospholipase C epsilon 1 gene (*PLCE1*) to be associated with DENV infection and severe dengue in a Vietnamese population^25^. To the best of our knowledge, our study is the first report showing an association of *MBL2* polymorphisms in the promoter and exon 1 regions including *MBL2* genotypes (− 550*L/H, MBL2* codon 54*), MBL2* diplotypes (*XA/XO*, *YA/XO*) and *MBL2* haplotypes (*LXPB*, *HXPA*, *XO*) with DENV infection and dengue in Vietnam.

In DENV infection, the complement system is consistently activated^26^. However, the frequencies of *MBL2* variants, which are associated with MBL levels, are not significantly different when comparing dengue patients and controls. In addition, the *MBL2 OO* genotype and the *MBL2 O* allele, which are involved both in regulation of MBL levels and the recognition of pathogens, differ between dengue patients and controls. The DENV envelope consists of E and M proteins with high-mannose glycans that are recognized and opsonized by MBL^16^. Although sufficient MBL levels and functions are required to neutralize DENV via complement-mediated mechanisms^15^, our results show that serum MBL levels do not differ significantly between healthy individuals, dengue and DWS patients. These results suggest that *MBL2* variants predominantly contribute to complement activation by recognizing DENV antigens, rather than regulate MBL serum levels.

The complement proteins C2, C4b, C5, C5a, and factor D are significantly modulated during clinical course of DENV infection, suggesting that activation of the complement system through alternative pathways might be more dominant in response to DENV infection and, thus, might contribute to dengue severity. This is supported by a previous study indicating the effect of alternative complement pathway deregulation on dengue severity via imbalance of factor D and factor H levels in DHF patients^27^. In addition, overactivity of the alternative complement pathway in the cellular microenvironment plays a crucial role in the pathogenesis of dengue, involving increased vascular permeability and plasma leakage^28^. Notably, the expression of the key regulators of the alternative complement pathway including factor H and factor B is induced by DENV infection in DENV-targeted cells^28^. Factor B is known as an activator of the alternative pathway and factor D is a serine protease that cleaves factor B and, thus, contributes to activation of the alternative pathway^29^. Deficiency of factor D has been shown to be associated with increased susceptibility to dengue infection^30^; factor D levels were decreased in dengue compared to healthy controls^27^. Similarly, the decrease of factor D levels, but not factor I, in dengue and DWS patients compared to healthy controls as well as their correlation with clinical parameters in both dengue and DWS suggests that lower levels of factor D are associated with an increased risk of DENV infection and disease severity.

Although in the present study C4b levels did not differ significantly between dengue patients and healthy individuals, higher C4b levels in DWS patients compared to dengue patients suggest a functional role of C4b in disease progression. The NS1 protein of flaviviruses has been shown to both interact with C4 to promote degradation of C4 to C4b and to bind to the C4b binding protein (C4BP) to inactivate C4b, and these interactions lead to reduced activation of the classical and the lectin pathway^31,32^. C2, C5, and C5a levels were significantly increased in dengue patients compared to controls and are highest in DWS patients, indicating that the complement system is strongly activated in order to respond to DENV infection. A previous study has shown that C5a does not only interact with DENV antigens in human hosts but also reduces DENV infection in mosquito cells^33^. Several host biomarkers including C5a and factor D levels have been suggested to support the progression of dengue^34^. Our data also indicate that the levels of C2, C4b, C5, C5a, C9, factor D and factor I are significantly associated with clinical parameters, especially with platelet counts, of dengue patients. Therefore, C2, C4b, C5, C5a, and factor D levels may be considered markers for progression of DENV infection and severity of the disease.

Although this study provides interesting results of the functional role of complement proteins, there are several limitations. The complement proteins were not determined at different time points of the clinical course and the activity levels of the complement system during progression of dengue were not assessed continuously. Other complement proteins such as C1, C3 and ficolins were not determined.

In conclusion, our study shows that *MBL2* polymorphisms are associated with dengue in a Vietnamese study group and that C2, C4b, C5, C5a and factor D levels are significantly modulated in dengue patients and during the progression of dengue. Our data point to the crucial role of the alternative complement pathway in response to DENV infection and disease severity.
